# Cardiovascular and renal oxidative stress-mediated toxicities associated with bisphenol-A exposures are mitigated by *Curcuma longa* in rats

**DOI:** 10.22038/AJP.2023.23367

**Published:** 2024

**Authors:** Temitayo Olabisi Ajibade, Oluwaseun Olanrewaju Esan, Israel Mark Osawere, Moses Olusola Adetona, Oluwasanmi Olayinka Aina, Odunayo Ibraheem Azeez, Ayobami Deborah Obisesan, Ademola Adetokunbo Oyagbemi, Olufunke Eunice Ola-Davies, Temidayo Olutayo Omobowale, Adebowale Benard Saba, Adeolu Alex Adedapo, Momoh Audu Yakubu, Oluwafemi Omoniyi Oguntibeju

**Affiliations:** 1 *Department of Veterinary Physiology and Biochemistry, Faculty of Veterinary Medicine, University of Ibadan, Nigeria*; 2 *Department of Veterinary Medicine, Faculty of Veterinary Medicine, University of Ibadan, Nigeria*; 3 *Department of Anatomy, Faculty of Basic Medical Sciences, University of Ibadan, Nigeria*; 4 *Department of Anatomy, Faculty of Veterinary Medicine, University of Ibadan, Nigeria*; 5 *Department of Veterinary Pharmacology and Toxicology, Faculty of Veterinary Medicine, University of Ibadan, Nigeria*; 6 *Department of Environmental & Interdisciplinary Sciences, College of Science, Engineering & Technology, COPHS,Texas Southern University, Houston, TX, USA*; 7 *Department of Biomedical Sciences, Faculty of Health and Wellness Sciences, Cape Peninsula University of Technology, Bellville 7535, South Africa*

**Keywords:** Bisphenol A, Curcuma longa, Rat, Antioxidant, Anti-inflammatory

## Abstract

**Objective:**

*Curcuma longa *Rhizome (CLR), due to its potent antioxidant phytochemical constituents, was investigated for its effects on bisphenol A (BPA)-induced cardiovascular and renal damage.

**Materials and Methods:**

Sixty rats were randomly selected, and grouped as control, BPA (100 mg/ kg), BPA and CLR 100 mg/kg, BPA and CLR 200 mg/kg, CLR 100 mg/kg, and CLR 200 mg/kg for 21 days. Oxidative stress indices, antioxidant status, blood pressure parameters, genotoxicity, and immunohistochemistry were determined.

**Results:**

Rats exposed to the toxic effects of BPA had heightened blood pressure, lowered frequency of micronucleated polychromatic erythrocytes, and decreased activities of antioxidant enzymes compared with rats treated with CLR. Moreover, administration of CLR significantly (p<0.05) lowered malondialdehyde content and reduced the serum myeloperoxidase activity. Immunohistochemical evaluation revealed significantly (p<0.05) increased expressions of cardiac troponin and Caspase 3 in the BPA group compared with the CLR-treated groups.

**Conclusion:**

*C. longa* ameliorated cardiotoxic and nephrotoxic actions of bisphenol-A via mitigation of oxidative stress, hypertension, and genotoxicity.

## Introduction

**Figure 1:**
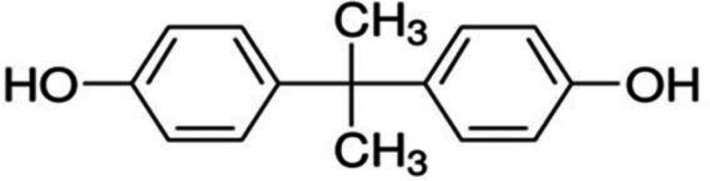
Schematic diagram illustrating chemical structure of bisphenol-A

Bisphenol A (BPA) is an inorganic environmental contaminant and endocrine disruptor found in water, air, and soil (Kang et al., 2006; Murata et al., 2018)** (**see Figure 1). In addition to the environmental sources of exposure, humans and animals are exposed via dietary packages containing high levels of BPA (Chen et al., 2016), with consequent toxicological manifestations on cardio-renal physiology (Gassman, 2017; Wu et al., 2021). In the cardiovascular system, BPA causes cardiac arrhythmia, myocardial infarction, and tachycardia by aggravating the formation of pro-oxidant molecules (Ola-Davies and Olukole, 2018), with consequent induction of structural and functional oxidative stress-mediated changes to deoxyribonucleic acid (Khodayar et al., 2018). Likewise, BPA promotes apoptosis, mitochondrial dysfunction, and inflammation in the renal tissue (Wu et al., 2021).

Medicinal plants such as *Asparagus officinalis*, *Adiantum capillus*, Ginger, *Pistacia integerrima*, *Vincetoxicum arnottianum*, and *Murraya koenigii* have been reported to positively modulate several mammalian physiological systems and ameliorate BPA-induced toxicities in rats (Yousaf et al., 2016; Kanwal et al., 2018; Mohammed et al., 2020). Moreover, antioxidant-rich phytochemical constituents of medicinal plants such as *Asparagus officinalis* have been reported to ameliorate oxidative stress and histopathological changes in the kidney of rats intoxicated with BPA (Poormoosavi et al., 2018). In the same vein, green tea extract and epigallocatechin gallate have been reported to be effective in preventing or reducing metabolic disorders induced by BPA due to their antioxidant and anti-inflammatory activities (Mohsenzadeh et al., 2021). *Curcuma longa* rhizome (CLR) Linn is a plant traditionally used as alternative therapy to conventional drugs for the management of diseases (Sultana et al., 2021). The biological activities of CLR for which it has been explored for the alternative management of diseases, include anti-inflammatory, anticancer, antioxidant, antibacterial, antimicrobial, antidiabetic, and immunomodulatory activities (Cárdenas-Garza et al., 2021; Mekonnen and Desta, 2021; Memarzia et al., 2021; Gururani et al., 2022, Ghasemi et al., 2022; Ghasemi et al., 2022). The antioxidant activity of curcumin, an important phytochemical constituent of CLR, reportedly ameliorated toxicant-induced organ derangements, including cardiac and renal toxicities (Ramkumar et al., 2021). The *in vivo* micronucleated polychromatic erythrocytes (MnPCEs) as a marker of genomic instability and DNA damage arising from environmental pollutants and some drugs such as BPA have been reported (Motto et al., 2020; Sommer et al., 2020; Bhilwade et al., 2014; Rocco et al., 2012). In view of the high risk of exposure to BPA from environmental sources, the present study was designed to investigate the modulatory effects of CLR against BPA-induced cardiovascular and renal toxicities. Also, we aimed to investigate the antioxidant potential of CLR against BPA-induced, oxidative stress-mediated deraignment of normal physiological functioning in Wistar rats. 

## Materials and Methods

**Experimental groupings**

Sixty adult male Wistar rats having weight range 120 - 150 grams were purchased and selected without prejudice to weight or size as follows: Groups A, control, B, BPA (100 mg/kg) only, C, BPA and 100 mg/kg CLR**,** D, BPA and 200 mg/kg of CLR, E, 100 mg/kg of CLR only, and group F, 200 mg/kg of CLR only. We chose the dosage of BPA from the previous study of Akintunde et al. (2020), while that of *C. longa *was adapted from the study of (Oridupa et al., 2021). The administration of BPA alone or together with CLR was done by oral gavage, for a period 3 weeks. We followed ethical regulations in accordance with national and institutional guidelines for the protection of animal welfare during experiments.

**Experimental plant**

The *Curcuma* *longa* rhizome purchased from different Markets in Ibadan and its environs within Oyo state, Nigeria were collected freshly and preserved at optimum temperature. The CLR were identified at the department of Botany, University of Ibadan and the voucher specimen number UIH-24571 was deposited in the herbarium. The fresh rhizomes (200 g) were rinsed, air dried and then grinded into fine powder. The powder was weighed and immersed in absolute ethanol (2.5 l) for two 48 hr with regular stirring. The mixture was thereafter filtered with muslin cloth and a Whitman filter paper producing the final filtrate, which was concentrated at 45^ᵒ^C with a rotary evaporator, dried in a temperature-controlled oven, and stored at 4^ᵒ^C until required for use. The final extract weighed 72.26 g, giving a percentage yield of 12.05%.

**Haemodynamic parameters**

The systolic and diastolic blood pressures and mean arterial pressure were determined non-invasively in conscious animals by using an automated blood pressure monitor (CODA SI, Kent Scientific Corporation, USA) on day 20 of the experiment. The readings were taken in the quiescent state, following animal acclimatization as previously documented by Omobowale et al. (2019).

**Serum and tissue preparation**

The rats were humanely handled, and about 3 ml of blood were collected into plain bottles and subjected to centrifugation at 4,000 revolution per minute for 15 min, to obtain the serum which was carefully decanted into clean bottles, and refrigerated at -4^o^C throughout the experimental period.

Following anaesthesia (87.5 mg/kg Ketamine/ 12.5 mg/kg Xylazine), the heart and kidney tissues were harvested from carefully dissected rats and weighed before homogenisation in twenty (20) volumes for the heart and ten (10) volumes for the kidneys of 0.1 M phosphate buffer, and subjected to cold centrifugation at 4,000 revolution per minute for 15 min to obtain post mitochondrial fraction (Oyagbemi et al., 2019). 

***In vivo*** **micronucleus assay technique**

The proximal ends of the femurs were carefully removed with a pair of scissors until a small opening to the marrow became visible. The femur was submerged in fetal calf serum and the marrow was flushed out gently by aspiration and flushing on glass slides. The marrow suspension was positioned on one end of a slide and spread by pulling the material behind polished cover glass held at an angle of 45^º^. Slides were fixed in methanol for 3-5 min; allowed to dry for 24 hr, stained with May-Gruenwald stain, and later with 5 % diluted Giemsa solution for at least thirty minutes. Slides were then rinsed in phosphate buffer for about 30 sec and in distilled water. Slides were then air-dried. The dried stained slides were then mounted in dibutylphthalate polystyrene xylene (DPX) with coverslips, and viewed under the microscope at X 100 magnification using oil immersion for the presence of micronucleated polychromatic erythrocytes (MNPCE). Scoring was done using a tally counter.

**Determination of** ***in vivo*** **antioxidant status and oxidative stress indices**

Markers of oxidative stress and antioxidant status such as glutathione (GSH) level were determined as described by Jollow et al. (1974). Superoxide dismutase (SOD) activity was determined as described by Misra and Fridovich (1972), with slight modification from our laboratory (Oyagbemi et al., 2019). The activity of glutathione peroxidase (GPx) was also measured according to the method of Beutler et al. (1963), while the activity of glutathione S-transferase (GST) was estimated by the method of Habig et al. (1974). Further, protein thiol (PSH) and non-protein thiol (NPSH) contents were determined as previously described by Ellman (1959).

The hydrogen peroxide (H_2_O_2_) production and serum myeloperoxidase (MPO) activities were estimated as reported by Wolff (1994) and Pulli et al. (2013), respectively. Vitamin C contents were quantified as previously described by Jacques-Silva et al. (2001). Malondialdehyde (MDA) content as an index of lipid peroxidation was quantified in the cardiac and renal tissues according to the method of Varshney and Kale (1990). The absorbance was estimated against blank at 532 nm. Lipid peroxidation index was calculated with a molar extinction coefficient of 1.56×10^5^/M/cm. Serum nitric oxide concentrations were measured spectrophotometrically at 548 nm according to the method of Olaleye et al. (2007). 

**Protein determination**

Tissue and serum protein concentrations were determined with Biuret method as previously described (Gornal et al., 1949). Potassium iodide was added to the reagent to prevent precipitation of Cu^2+^ ions as Cuprious oxide.

**Histopathology**

The heart and kidneys were collected in 10% formalin saline buffer for proper fixation. These tissues were processed and embedded in paraffin wax. Sections of 5 – 6 µm in thickness were made and stained with Haematoxylin and Eosin for histopathological examination (Drury et al., 1976)

**Immunohistochemistry**

The immunolocalization of cardiac troponin (CTnI) and caspase 3 was determined according to the method of Oyagbemi et al. (2019), with cardiac and renal tissues fixed using 10% paraformaldehyde, embedded in paraffin, and sectioned at a thickness of 5 μm. The antigen retrieval was carried out in 10 mM citrate (6.0), in microwave oven. Goat serum (E-1R-R217A) was used to prevent specific binding. Cardiac antitroponin I (E-AB-70275: 1:500) and anticaspase 3 polyclonal antibody (E-AB-13815:1:200) were used for incubation for 2 hr, at room temperature, and thereafter, probed with secondary antibody (E-1R-R217B). Substrate diaminobenzidine (DAB) was added to improve immunopositive reactions. 

**Data analysis**

All values were expressed as mean±standard deviation (SD) and the test of significance between two groups was estimated with Student’s t-test. The One-Way Analysis of Variance (ANOVA) with Turkey’s post-hoc test was also carried out with p-values<0.05 considered statistically significant. 

## Results

**Effects of bisphenol-A and** ***C. longa*** **on blood pressure parameters**

**Figure 2:**
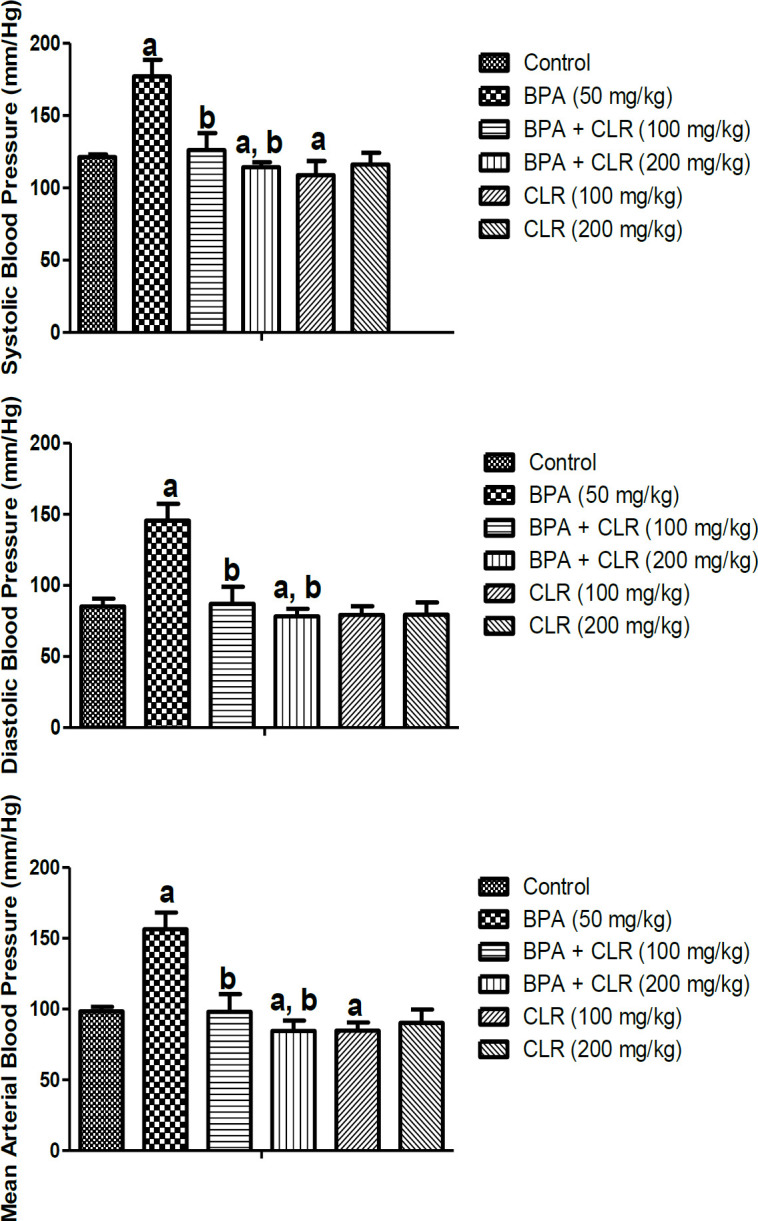
Effects of BPA and CLR on blood pressure parameters; systolic, diastolic and mean arterial blood pressure. mg/kg, Bisphenol-A + CLR 200 mg/kg, CLRSuperscript (a) indicates a significant difference when compared to the control while (b) indicates a significant difference when compared to Bispheol A alone. Values are presented as Mean±SD (n=5).

Bisphenol-A caused a significant (p<0.05) increase in systolic, diastolic, and mean arterial pressures in the rats exposed to BPA compared with the control group. Treatment groups BPA + 100 mg/kg CLR and BPA + 200 mg/kg CLR exhibited a significantly lowered parameters of blood pressure relative to the untreated group as indicated in Figure 2.

**Effects of Bisphenol A and** ***C. longa*** **on micronucleated polychromatic erythrocyte (MPCEs)**

**Figure 3:**
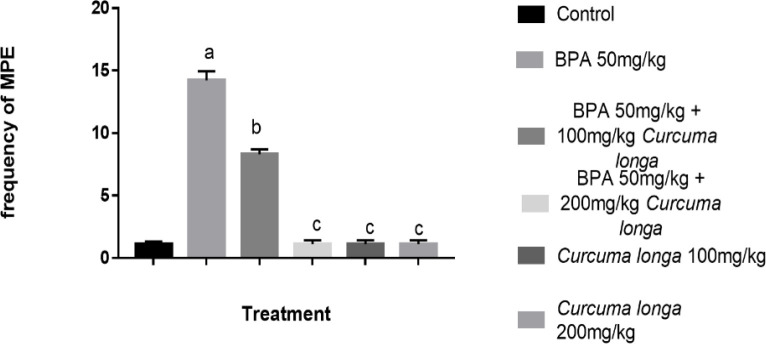
Effects of BPA and CLR on micronucleated polychromatic erythrocyte of Wistar albino rats. Superscript (a) indicates a significant difference when compared to the control while (b) indicates a significant difference when compared to Bispheol A alone. Values are presented as Mean±SD (n=5).

As highlighted in Figure 3, Bisphenol-A caused increased frequency of MPCEs in comparison with the BPA + 100 mg/kg CLR and BPA + 200 mg/kg CLR groups (Figure 3). The anti-genotoxic effect of CLR was demonstrated as indicated. with a significant reduction in the frequency of MPCEs in comparison to the BPA group. It is worth noting that 200 mg/kg of CLR normalized the frequency of MPCEs to near the control values (Figure 3). 

**Effects of** ***C. longa*** **and Bisphenol-A on organo-somatic index**

BPA administration caused a significant (p<0.05) reduction in body weight when compared with the control (Table 1). Similar observation was found in rats administered with BPA +100 mg/kg CLR relative to rats administered only with BPA (Table 1). However, there was an appreciable increase in the body weights of rats administered with BPA +200 mg/kg *C. longa, *100 mg/kg CLR and 200 mg/kg CLR (Table 1). 

**Cardio-renal oxidative stress indices and antioxidant status**

Bisphenol-A intoxication caused a significant (p<0.05) decrease in reduced glutathione (GSH) content, superoxide dismutase (SOD), glutathione S-transferase (GST) and glutathione peroxidase (GPx) activities, in cardiac and renal tissues, compared with the control group (Tables 2 and 3). Similarly, there was a noticeable improvement in GSH content of cardiac tissues which was statistically significant in rats administered with BPA +100 mg/kg CLR relative to rats administered only with BPA (Table 2). However, we also recorded a significant (p<0.05) decrease in GSH and vitamin C contents, and the activities of SOD, GST and GPx of rats intoxicated with 50 mg/kg of BPA when compared to the control (Table 3). Bisphenol-A toxicity caused a significant (p<0.05) increase in cardiac hydrogen peroxide (H_2_O_2_) generation, malondialdehyde (MDA) content and significantly decreased protein thiol (PSH) and non-protein thiol (NPSH) contents, respectively, when compared to the control group. 

**Table 1:** Effects of *Curcuma longa* and Bisphenol-A on body and organ weights in Wistar rats

**Parameters**	**Control**	**Bisphenol-A (BPA) 50 mg/kg**	**BPA +100 mg/kg ** ***Curcuma longa***	**BPA +200 mg/kg ** ***Curcuma longa***	**100 mg/kg ** ***Curcuma longa***	**200 mg/kg ** ***Curcuma longa***
Final Body Weight (g)	139.8±18.60	136.0±11.40^a^	134.0±16.73^ b^	146.0±5.50^ b^	154.0±5.50^ b^	164.0±11.40^ b^
Initial Body weight (g)	98±8.36	102±13.02	104±11.40	114±8.94	112±10.95	114±8.94
Heart (g)	0.45±0.06	0.43±0.03	0.37±0.03^a, b^	0.30±0.03^a, b^	0.29±0.03^a^	0.31±0.01^a^
Kidney (g)	0.91±0.07	0.89±0.04	0.77±0.08^a, b^	0.55±0.08^a, b^	0.59±0.07^a^	0.65±0.05^a^

Superscript (a) indicates a significant difference when compared to the control while (b) indicates a significant difference when compared to Bispheol A alone at p<0.05 (n=5). Superscripts denote statistical difference across all groups in each row. Values are presented as mean±standard deviation.

**Table 2:** Effects of *Curcuma longa* and Bisphenol-A on cardiac antioxidant defense system in Wistar rats

**Parameters**	**Control**	**Bisphenol-A (BPA) 50 mg/kg**	**BPA +100 mg/kg ** ***Curcuma longa***	**BPA +200 mg/kg ** ***Curcuma longa***	**100 mg/kg ** ***Curcuma longa***	**200 mg/kg ** ***Curcuma longa***
GSH (µmole/mg protein)	97.10±4.26	85.17±6.02^a^	82.60±3.29^a^	104.4±4.50^a,b ^	117.19±8.97^a^	93.43±7.85^a^
SOD (% inhibition)	26.52±2.55	24.88±3.34	23.30±3.72	22.77±2.31^a^	21.48±0.93^a^	22.11±2.20^a^
GST (CDNB-GSH complex formed/min/mg protein)	22.41±1.69	18.10±2.76^a^	17.65±1.10^a^	16.80±1.20^a^	19.04±3.60^a^	16.40±0.84^a^
GP_X _(units/mg protein)	27.30±2.50	22.86±1.64^a^	22.64±1.20^a^	23.15±1.25^a^	23.32±3.23^a^	24.36±2.41^a^
VIT C (µmol/mg protein)	13.37±1.60	12.93±1.16	13.12±1.45	11.98±1.34	12.60±0.99	12.82±1.52

Superscript (a) indicates a significant difference when compared to the control while (b) indicates a significant difference when compared to Bispheol A alone at p<0.05 (n=5). Superscripts denote statistical difference across all groups in each row. Values are presented as mean±standard deviation.

**Table 3:** Effects of *Curcuma longa* and Bisphenol-A on renal antioxidant defense system in Wistar rats

**Parameters**	**Control**	**Bisphenol-A (BPA) 50 mg/kg**	**BPA +100 mg/kg ** ***Curcuma longa***	**BPA +200 mg/kg ** ***Curcuma longa***	**100 mg/kg ** ***Curcuma longa***	**200 mg/kg ** ***Curcuma longa***
GSH (µmole/mg protein)	82.96±9.02	86.43±7.28	94.53±11.35	80.12±3.03	82.90±3.92	92.82±14.72
SOD (% inhibition)	35.30±3.98	12.66±1.87^a^	38.57±4.00^b^	30.66±1.33^a, b^	39.55±7.00	28.97±4.60^a^
GST (CDNB-GSH complex formed/min/mg protein)	23.22±4.12	12.13±2.88^a^	32.77±6.17^a,b^	27.63±5.30^b^	36.90±5.77^a^	35.76±3.98^a^
GP_X _(units/mg protein)	36.07±5.92	40.70±5.84	46.00±6.41^a^	43.85±4.06^a^	48.04±5.96a	53.35±5.16a
VIT C (µmole/mg protein)	1.64±0.33	1.41±0.03	1.87±0.89	1.68±0.23^b^	1.58±0.11	1.63±0.14

Superscript (a) indicates a significant difference when compared to the control while (b) indicates a significant difference when compared to Bisphenol A alone at p<0.05 (n=5). Superscripts denote statistical difference across all groups in each row. Values are presented as mean ± standard deviation.

Interestingly, CLR co-treatment with BPA significantly lowered H_2_O_2_ generation, and malondialdehyde (MDA) content, with a concomitant significant increase in PSH and NPSH (Tables 4 and 5).

**Effects of Bisphenol-A and** ***Curcuma longa*** **on Myeloperoxidase (MPO)**

Myeloperoxidase (MPO) as a marker of inflammation was also assessed in this study. The BPA intoxication caused a significant (p<0.05) increase in the MPO values of rats exposed to BPA only compared to the control and other treatment groups (Table 6). The anti-inflammatory property of CLR at 200 mg/kg showed significant reduction (p < 0.05) in MPO activities in comparison to BPA intoxicated group (Table 6).

**Table 4:** Effects of *Curcuma longa* and Bisphenol-A on markers of oxidative stress in the heart of Wistar rats

**Parameters**	**Control**	**Bisphenol-A (BPA) 50 mg/kg**	**BPA +100 mg/kg ** ***Curcuma longa***	**BPA +200 mg/kg ** ***Curcuma longa***	**100 mg/kg ** ***Curcuma longa***	**200 mg/kg ** ***Curcuma longa***
H_2_O_2_ (µmole formed/min/mg protein)	15.40±0.29	16.44±0.55^a^	12.25±0.33^ a, b^	15.61±1.77^ b^	13.11±0.59^ a^	13.11±2.36^ a^
PSH (µmole/mg protein4	93.02±13.23	78.60±9.25^a^	67.03±3.05^a, b^	78.43±9.70^a^	95.31±7.93	90.95±9.24
NPSH (µmole/mg protein)	16.09±2.12	14.97±2.90	9.83±1.97^a, b^	13.96±0.71	16.18±1.60	18.10±3.15
MDA (µmole formed/mg protein)	2.67±0.61	5.12±0.53^a^	3.46±0.70^a, b^	2.46±0.80^b^	2.80±0.39	2.75±0.76

Superscript (a) indicates a significant difference when compared to the control while (b) indicates a significant difference when compared to Bisphenol A alone at p<0.05 (n=5). Superscripts denote statistical difference across all groups in each row. Values are presented as mean±standard deviation. Abbreviations: H_2_O_2_ hydrogen peroxide generation (µmol/min/mg protein), MDA malondialdehyde (µmol of MDA formed/mg protein), PSH Protein thiol (µmole/mg protein), NPSH non protein thiol (µmole/mg protein)

**Table 5:** Effects of *Curcuma longa* and Bisphenol-A on markers of oxidative stress of the kidney of Wistar rats

**Parameters**	**Control**	**Bisphenol-A (BPA) 50 mg/kg**	**BPA +100 mg/kg ** ***Curcuma longa***	**BPA +200 mg/kg ** ***Curcuma longa***	**100 mg/kg ** ***Curcuma longa***	**200 mg/kg ** ***Curcuma longa***
H_2_O_2_ (µmole formed/min/mg protein)	149.65±15.43	185.71±14.00^a^	113.76±13.10^a,b^	125.29±6.63^a, b^	118.79±15.20^a^	135.39±15.91
PSH (µmole/mg protein)	9.75±0.27	8.79±0.42^a^	8.26±0.15^a, b^	1.37±4.10^a, b, c^	1.42±0.31^a^	1.44±0.42
NPSH (µmole/mg protein)	97.48±10.6	90.85±8.48	115.83±17.13^a,b^	140.56±12.43^a,c^	170.10±18.65^a^	113.7±5.34 ^a^,^e^
MDA (µmole formed/mg protein)	23.82±3.96	18.41±1.77^a^	17.33±2.83^a^	16.55±2.16^a^	18.60±2.56^a^	17.91±3.48^a^
						

Superscript (a) indicates a significant difference when compared to the control while (b) indicates a significant difference when compared to Bisphenol A alone at p<0.05 (n=5). Superscripts denote statistical difference across all groups in each row. Values are presented as mean±standard deviation. Abbreviations: H_2_O_2_ hydrogen peroxide generation (µmol/min/mg protein), MDA malondialdehyde (µmol of MDA formed/mg protein), PSH Protein thiol (µmole/mg protein), NPSH non protein thiol (µmole/mg protein)

**Table 6:** Effects of *Curcuma longa* and Bisphenol-A on serum myeloperoxidase activity in Wistar rats

**Parameters**	**Control**	**Bisphenol-A (BPA) 50 mg/kg**	**BPA +100 mg/kg ** ***Curcuma longa***	**BPA +200 mg/kg ** ***Curcuma longa***	**100 mg/kg ** ***Curcuma longa***	**200 mg/kg ** ***Curcuma longa***
MPO (unit/mg protein)	6.90±1.40	10.91±2.16^a^	6.12±1.01^b^	3.63±1.54^a, b^	6.68±0.81	6.46±0.25

Superscript (a) indicates a significant difference when compared to the control while (b) indicates a significant difference when compared to Bisphenol A alone at p<0.05 (n=5). Superscripts denote statistical difference across all groups in each row. Values are presented as mean±standard deviation.

**Immunohistochemistry of cardiac Caspase 3**

**Figure 4:**
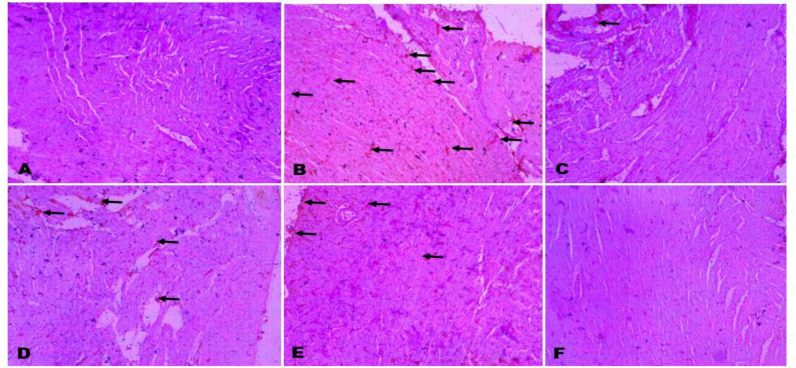
The immunohistochemistry of cardiac caspase 3. A (control), B (BPA), C (BPA + CLR 100 mg/kg), D (BPA + CLR 200 mg/kg), E (CLR 100 mg/kg), and F (CLR 200 mg/kg). Slides stained with high definition Haematoxylin. Magnification at X 100

The BPA only showed the highest expression of cardiac Caspase 3 compared to all other groups. However, BPA + 100 mg/kg CLR and BPA + 200 mg/kg CLR showed a lower expression of cardiac caspase compared to the BPA only group. CLR crude extract also showed mild expression of cardiac caspase 3 (black arrows) as indicated in Figure 4. 

**Immunohistochemistry of cardiac Troponin I**

**Figure 5:**
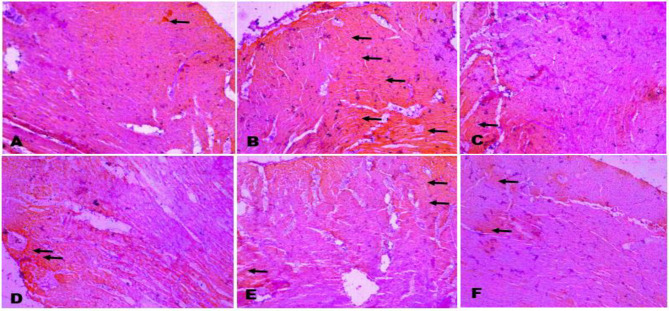
The immunohistochemistry of cardiac troponin I. A (control), B (BPA), C (BPA + CLR 100 mg/kg), group D (BPA + CLR 200 mg/kg), Group E (CLR 100 mg/kg), and group F (CLR 200 mg/kg). Slides stained with high definition Haematoxylin. Magnification at X 100

As shown in Figure 5, BPA toxicity gave the highest expression of cardiac troponin as compared to all other groups. However, co-administration of BPA with CLR gave a lower expression of cardiac troponin compared to the BPA only group. Also, administration of CLR alone showed mild expression of cardiac troponin (Figure 5). 

**Histopathological changes**

**Figure 6:**
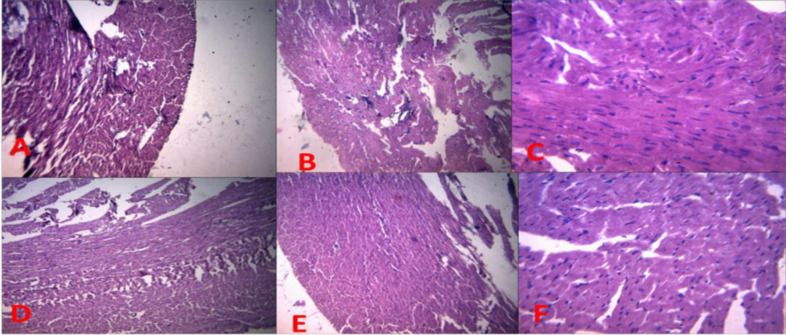
The histopathology of the heart showing the effect of BPA with or without CLR in Wistar albino rats. A (control), B (BPA), C (BPA + CLR 100 mg/kg), D (BPA + CLR 200 mg/kg), E (CLR 100 mg/kg), and F (CLR 200 mg/kg). Slides stained with high definition Haematoxylin and Eosin. Magnification at X 100

**Figure 7:**
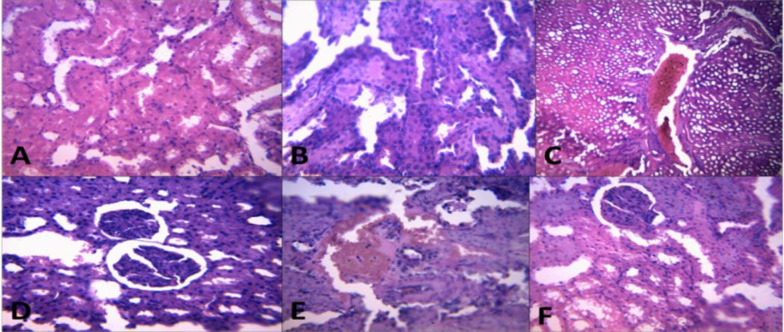
The histopathology of the heart showing the effect of Bisphenol A with or without Curcuma longa in Wistar albino rats. A (control), B (BPA), C (BPA + CLR 100 mg/kg), D (BPA + CLR 200 mg/kg), E (CLR 100 mg/kg), and F (CLR 200 mg/kg). Slides stained with high definition Haematoxylin and Eosin. Magnification at X 100.

In the cardiac tissues, BPA induced focal loss of myofibre striation, degeneration, and inflammatory cell infiltration. These lesions were absent in the CLR treated groups (Figure 6). In the kidneys of BPA group, mild to severe haemorrhages were observed in the renal interstitium; while rats co-administered with BPA + 100 mg/kg CLR and 200 mg/kg CLR revealed less visible pathological lesions as shown in Figure 7. 

## Discussion

The utilization of medicinal plant products for management of ill-health of humans is commonplace in the developing world due to affordability, perceived beneficial effects and reduced side effects (Laar et al., 2021; Ashima et al., 2021). Our results showed that CLR ameliorated hypertension associated with BPA toxicity as compared to the control. We recorded a significant reduction in systolic, diastolic, and mean arterial pressures in rats treated with CLR, indicative of anti-hypertensive action of CLR. This observation of antihypertensive effect for CLR corroborates earlier report of Hasimun et al. (2021) who reported CLR as a vasodilator with potent antihypertensive effect mediated by improved vascular elasticity and potentiation of nitric oxide bioavailability. The anti-hypertensive action of CLR might be correlated with anti-inflammatory and antioxidant activity of CLR as earlier mentioned. 

Also, in the present study, BPA-induced oxidative stress, seen as significant increases in the pro-oxidant but decreases in the systemic antioxidants, were mitigated by CLR. This observation underscores the widely acclaimed potent antioxidant effect of CLR which may have contributed significantly to the mitigation of BPA toxicities and hypertension seen in rats in the present study. Oxidative stress has been frequently implicated in the pathogenesis of hypertension, and its amelioration usually correlates with normotensive status and normal vascular activities. The modulation of the vasomotor system involves reactive oxygen species as mediators of vasoconstriction induced by angiotensin II, endothelin-1 and urotensin-II, and the bioavailability of nitric oxide (NO), a major vasodilator, is highly dependent on the redox status (Rodrigo et al., 2011). Moreover, there is a positive correlation between the impairment of the antioxidant capacity and blood pressure elevation, thus underlying the relevance of antioxidant-rich molecules for the treatment of hypertension (Gonzalez et al., 2014). Therefore, mitigation of oxidative stress and normotensive status observed in the present study may be linked to the high constituents of antioxidant phytochemicals of CLR, as earlier reported (Braga et al., 2018). 

From our study, we discovered that BPA intoxication significantly increased the frequency of micronucleated polychromatic erythrocytes (MnPCEs). Previous studies reported that increased frequency of MnPCEs has been positively correlated to genomic instability and damage to DNA (Motto et al., 2021; Sommer et al., 2020). Also, research findings elsewhere revealed that exposure to BPA caused DNA damage and mutagenicity (Jalal et al., 2017; Gassman et al., 2016; Pfeifer et al., 2015; Iso et al., 2006). Our finding agrees with earlier reports on the genotoxic and mutagenic effect of BPA intoxication. We found it exciting to report that rats intoxicated with BPA and treated with CLR had lower values of MnPCEs, which is indicative of antigenotoxic property of CLR in a dose-dependent manner with the 200 mg/kg of CLR being more potent. Hence, our findings are in line with previous report of antigenotoxic and non-mutagenic effects CLR (Hosseini et al., 2018; Soleimani et al., 2018; Mendonça et al., 2015).

The high immunohistochemical expression of cardiac troponin and caspase 3 indicates involvement of myocardial destruction and apoptosis in BPA-induced cardiotoxicity. High sensitive biomarkers such as cardiac troponins I (cTnI) and T (cTnT) have been shown to be highly sensitive and specific markers of myocardial cell injury (Hammarsten et al., 2018). Exposure to 50 mg/kg of BPA for 8 weeks was reported to cause morphological and structural changes in the rat myocardium in the form of vacuolated myocytes, focal loss of myofibrils, and distorted mitochondria and tubular system (Abd El-Haleem et al., 2012). Furthermore, modulation of estrogen receptor signaling, alteration of cardiac Ca^2+^-handling protein expressions, ion channel inhibition/activation, oxidative stress and free radical formation, and genome/transcriptome modifications have been implicated in BPA-toxicity (Bahey et al., 2019). Therefore, the attenuated immunohistochemical expressions of cardiac troponin and caspase 3, as observed in rats administered with CLR, in this study, strongly suggest anti-apoptotic mechanism of the plant in the mitigation of toxic mechanisms associated with BPA exposure. This observation corroborates an earlier report on the reversal of neuronal apoptosis by CLR in sevoflurane-induced toxicity via the p-ERK1/2 pathway (Yu et al., 2021). Moreover, mitochondriaderived reactive oxygen species were reported to be reduced in CLR extract treated SH SY5Y cells with the downregulation of mRNA expression levels of p53, Bcl 2 associated X protein and caspase 3 (Ma et al., 2017), further underlining the positive modulatory role of CLR in the suppression of apoptotic mechanisms in toxicological processes. 

In conclusion, *Curcuma longa* ameliorated the cardiotoxic and nephrotoxic effects of bisphenol A in a dose-dependent manner. Therefore, medicinal preparations containing *C. longa* could play beneficial roles in the treatment/management of toxicities associated with environmental or dietary bisphenol A exposure. 
